# Cost Analysis of Selected Radiotherapeutic Modalities for Prostate Cancer Treatment—Czech Republic Case Study for the Purposes of Hospital Based HTA

**DOI:** 10.3390/healthcare9010098

**Published:** 2021-01-19

**Authors:** Petra Hospodková, Tomáš Husár, Barbora Klíčová, Lucie Severová, Karel Šrédl, Roman Svoboda

**Affiliations:** 1 Department of Economic Theories, Faculty of Economics and Management, Czech University of Life Sciences Prague, 165 00 Prague, Czech Republic; petra.hospodkova@fbmi.cvut.cz (P.H.); severova@pef.czu.cz (L.S.); sredl@pef.czu.cz (K.Š.); 2 Department of Biomedical Technology, Czech Technical University in Prague, 272 01 Kladno, Czech Republic; Husar.Tom7@seznam.cz (T.H.); barbora.klicova@fbmi.cvut.cz (B.K.)

**Keywords:** 3D-CRT, activity based costing, IMRT, prostate cancer, sensitivity analysis

## Abstract

This study aims to calculate the costs of prostate cancer radiotherapy in a regional hospital Department of Radiation Oncology equipped with Three-Dimensional Conformal Radiation Therapy (3D-CRT) and Intensity Modulated Radiation Therapy (IMRT) Volumetric Arc Therapy (VMAT) radiation technology, using activity based costing (ABC), and to compare the costs of both methods at the level of component treatment process activities and with respect to insurance reimbursements. The costing was performed based on a sample of 273 IMRT VMAT patients and 312 3D-CRT patients in a regional hospital in the period from 2018 to 2019. The research has highlighted the necessity to place emphasis on factors that may skew the costing results. The resulting output has been supplemented by a sensitivity analysis, whereas the modeled parameter is represented by the time required for one patient fraction on a linear accelerator and the time the Radiology Assistant needs to prepare the complete radiation plan as part of radiotherapy planning. Moreover, the effects of the received grant, in the form of calculated write-offs, are also considered. The case study uses the example of radiotherapy to demonstrate the potential of ABC and suggests considering the application of this method as an effective management tool for cost and economic evaluation as part of comprehensive hospital assessment under the Hospital-Based Health Technology Assessment (HB-HTA) initiative.

## 1. Introduction

Recently, a growing interest in Hospital-Based Health Technology Assessment (HB-HTA) methods has been observed, since it can provide important support for decision-making in the area of purchasing, implementation and/or disinvestment of technologies or interventions in hospitals [1,2]. In modern competitive reimbursement environments, providers and policy makers are looking for cost-accounting solutions capable of informing process improvement and meeting the expectations of cost-control policies [3]. Ritrovato et al. [4] claim that HB-HTA is able to guarantee that all hospital economic, instrumental and human resources will be used and allocated with efficacy, efficiency, and economic criteria, ensuring high quality healthcare assistance. HB-HTA is considered to be a useful tool that facilitates faster and earlier decision-making and improves the effectiveness of accepted healthcare technologies at the hospital level, as well as patient safety.

Activity based costing (ABC) appears to be a suitable management tool for these purposes [5]. It provides a structured approach to analyzing activities, costing services, reducing costs and improving quality [6]. While the use of ABC in healthcare facilities has been evident for several years, its application is geared more towards cost management at the cost-center level rather than towards cost analysis of a specific diagnosis (or a group of diagnoses). The issue of healthcare system efficiency would be best addressed by a periodic and uniform evaluation of actual medical treatment costs, as the state healthcare system needs to efficiently allocate limited financial resources [7], which has now been included in the ongoing DRG (Diagnosis-related group) restart initiative in the Czech Republic. On the other hand, at HB-HTA level, healthcare providers should be able to draw a comparison between intervention results and costs in time and have their own overview at their disposal. The amount of insurance reimbursements does not always correspond to the actual costs of diagnosis, implying that knowledge of the resulting balance (reimbursement vs. actual costs) at the level of a diagnosis, or a group of diagnoses, can provide a valuable basis for further decision-making at the hospital management level. In the Czech Republic, profitability at the level of selected diagnoses is currently studied by Popesko et al. [8], for example; however, more extensive research with respect to radiotherapy is yet to be done.

The incidence of prostate cancer varies dramatically across geographic locations. Nearly 70% of newly diagnosed patients reside in developed countries. The highest incidence of this disease has been observed in Australia, New Zealand, North America, and in Northern and Western Europe. Southern Asia is currently a region with the lowest incidence of prostate cancer, which has, however, been on a sharp increase over the past 20 years [9]. Since 1990, the continuously rising incidence has to a considerable extent been due to the introduction of prostate-specific antigen (PSA) testing, which allows current oncology to diagnose the disease in its early stages. While the exact mechanism behind the development of the disease is not fully understood, the constant risk factors likely to cause its occurrence and progress are generally known [10]. These factors include the ever-increasing average age of the population, as its incidence is most prevalent in older age groups with a median age of 75, as well as a positive family history, black race and genetic predisposition. Determining the exact environmental carcinogens is difficult; however, available evidence points to eating habits, obesity, and sexually transmitted diseases, which may be the initiators of prostate inflammation and subsequent development of the disease. As screening is expected to have a strong potential for reducing the mortality rate, monitoring the global epidemiological situation is of paramount importance [11].

With the introduction of screening using PSA testing, it is now possible to monitor the disease in its very early stages and detect small and low-risk prostate cancers that do not otherwise manifest clinically in the patient’s day-to-day life. PC patients currently have three main treatment options to choose from. The therapeutic approach to localized low-to-medium risk prostate cancer involves a conservative method of active surveillance. This method consists of regular patient check-ups, transrectal ultrasound (TRUS), a PSA blood test about every six months and tumor tissue biopsies done at 18-month intervals. In this way, curative treatment is delayed until the patient meets the criteria for classifying the progression of the disease [12].

If the disease progresses or if active surveillance is impossible or even undesirable, other options for curative treatment will be considered. In current oncological practice, external beam radiotherapy, along with interstitial brachytherapy and radical prostatectomy, is one of the principal methods of curative treatment for localized and locally advanced prostate cancer. External beam radiation therapy (EBRT) is the most common type of radiation therapy used for cancer treatment, while the linear accelerator (LU, LINAC) is the most frequently used irradiator [13].

Implementation of the selected treatment strategy is preceded by a series of mandatory staging examinations, including digital rectal examination (DRE), TRUS, PSA testing, prostate biopsy, a lesser pelvis CT (computed tomography) scan for pelvic node examination, MR examination of the abdomen, and pelvis to determine the T stage, or a combination of both modalities. A liver ultrasound scan and an X-ray of the lungs in patients with PSA > 20 ng/mL is also required to rule out generalization. A surgical report must be issued for all patients who undergo surgery. Bone scintigraphy should also be used in the staging of patients with suspected distant metastases (with PSA > 10 ng/mL). Appropriate treatment is then recommended based on prognostic factors. The extent of the disease (staging) is determined based on TNM (classification system of malignant tumours), histology including the Gleason Score, the initial level of increased PSA values and the dynamics of changes in the PSA level, grouping the patients into the following risk categories according to Table 1 [14,15]:

Adjuvant irradiation of the prostate bed is recommended for patients with risk factors relating to RAPE (pT3a extraprostatic extension, pT3b seminal vesicle infiltration, positive resection margin, and detectable PSA). The occurrence of pelvic lymph node metastases is also a negative factor in the prognosis, in which case it would be appropriate to apply external irradiation in conjunction with long-term androgen deprivation therapy (neoadjuvant/concomitant/adjuvant).

Both of the radiotherapy techniques being assessed (3DCRT and MRT VMAT) can deliver highly conformal radiation and allow for the application of high radiotherapeutic doses to destroy the tumor tissue while sparing the surrounding vital organs, which in the case of prostate cancer include mainly the urinary bladder, rectum, small intestinal loops and femur heads.

Prostate cancer patients treated with hypofractionated accelerated radiotherapy (HART), using the IMRT VMAT method, receive the overall treatment within a shorter period of time. Moreover, the toxicity associated with prostate cancer treatment is effectively reduced, resulting in a lower incidence of both acute and late adverse responses to radiation. The reduced occurrence of gastrointestinal toxicity is amply supported by the respective study [16].

Globally, prostate cancer (“PC”) is the second-most frequently diagnosed oncological disease in men, while, on the imaginary scale, it is the fifth-leading cause of cancer-related death in males. It accounts for approximately 6.6% of all oncological deaths. Prostate cancer globally comprises 15% of all oncological diseases, imposing a significant burden on the public healthcare system [17].

Developments in radiotherapeutic technology have allowed for higher radiation conformity of an irregular target volume and dose escalation, which has a measurable impact on treatment results [18,19,20,21].The recent changes in PC radiotherapy consisted mainly in the transition of fractionation modes towards hypofractionated accelerated radiotherapy, which can only be made available to patients via Intensity Modulated Radiation Therapy (IMRT), more advanced forms of conformal radiotherapy. Application of Three-Dimensional Conformal Radiation Therapy (3D-CRT) to PC has been on the wane, as it no longer benefits the patients as much as IMRT. The current global trend is to expand the next development stage in radiotherapy via modulated irradiation beam intensity, including rotational techniques such as Volumetric Arc Therapy (VMAT), which enable high-precision irradiation beam modulation during one gantry rotation, thereby accelerating the entire treatment process, while exposing the patient to less radiation [22].

Hypofractionation modes contribute to both reducing the duration of radiotherapy and mitigating the toxic effects of PC therapy, as well as curtail the occurrence of the acute and delayed side effects of radiation. Numerous results validating the advantages of IMRT have already been presented in a wide range of randomized studies. For example, Ślosarek [23] has demonstrated in his study that with the same dose coverage to target volume the total dose absorption in patients is lowest when using IMRT/VMAT with photon-beam energy of 20 MV. Meanwhile, Sutani et al. [24] also confirmed that with the same dose coverage to target volume, the total dose absorption in patients is lowest when using IMRT/VMAT, showing a lowered risk of chronic rectal toxicity in PC patients when compared to 3D-CRT. Bauman et al. [25] obtained essentially similar results in his study. The decrease in the risk of late gastrointestinal toxicity is probably due to better dose distribution in space, because as the irradiation beam intensity during radiation is modulated, IMRT allows for accurate copying of the target volume shape with a high dose and minimum dose stress on the rectum. The study carried out by Yong et al. [16] further confirm the decreased incidence of gastrointestinal toxicity as mentioned above.

IMRT has seen its use in the treatment of tumor diseases via radiotherapy increase severalfold in the past 20 years. In the Czech Republic, the proportion of each method applied to the treatment of PC is unknown; however, given the required components and the precise technical specifications of individual radiotherapy clinics, all of these facilities can be assumed to possess the capabilities necessary to provide IMRT-based treatment.

It goes without saying that the introduction of these novel methods as standard for the treatment of tumor diseases is associated with substantially higher costs. The clinical operation and technical maintenance of the state-of-the-art IMRT linear accelerator components is financially challenging, but is should be noted that a portion of these costs would be compensated for by the improved treatment results, reduced treatment time and lower costs for the treatment of complications related to gastrointestinal toxicity. A study conducted by Schroeck et al. [26] indicate that despite IMRT being, in most cases, more expensive from the viewpoint of health insurance companies, this approach is generally perceived as more cost-effective. The higher costs of IMRT as well as the similarity of both techniques in terms of preparation and delivery of a curative radiotherapy dose to the target volume is also evidenced in a study by Yong et al. [16], determining the difference between IMRT and 3D-CRT at 1019 USD. However, Yong et al. [16] again emphasize the benefit of reduced toxicity and conclude that IMRT is a more cost-effective technique. When applying IMRT to patients with clinically localized PC, the QALY (quality-adjusted life-year) score stood at 0.023, which is equivalent to eight days lived in perfect health [18]. Carter et al. [19] calculated that this represents total savings of approximately 1.1 million USD per 1000 patients. Patients treated with IMRT ultimately received more QALYs than patients who underwent 3D-CRT treatment, corresponding to approximately 20 QALYs gained per 1000 patients treated. IMRT was further shown to have undeniable benefits in terms of improvement in treatment efficiency and lower toxicity, yielding also a reduction in total (long-term) costs. As the publications referenced above suggest, the positive clinical effect is irrefutable.

In order to obtain an accurate estimate of the total costs of radiotherapy treatment of patients with the C61 diagnosis, the second most common oncological disease, right after breast cancer in women, hospitals would do well to monitor the flow of both direct and indirect costs across the activities conducted as part of the entire process of radiotherapy in relation to both irradiation technologies to avoid inaccurate conclusions. For this, the ABC method, which is used widely across industries, appears to be the ideal tool.

Application of the ABC method to the healthcare sector requires that all specifics involved be considered. These specifics are generally defined by Popesko [27], for example, who brings attention to the issue with setting up the whole system in terms of obtaining relevant input data. While Drury [28] suggests dividing ABC into four phases: identifying the major activities, assigning costs to cost pools/cost centers for each activity, determining the cost driver for every activity and assigning the costs of activities to products, Lievens et al. [29] point out the necessity to also factor in specific radiotherapeutic steps and take them into account in the overall design of the model. For example, Van de Werf et al. [30] propose a three-step ABC model that includes time consumption as one of the factors in assigning treatment-related costs.

There is small number of studies that show how to monitor the real cost of diagnosis in healthcare facilities, and which also summarize the factors that have a significant effect on the overall calculation result, or diminish the informative value of the result.

This study aims to determine the costs of prostate cancer radiotherapy in a regional hospital Department of Radiation Oncology equipped with 3D-CRT and VMAT radiation technology, using the ABC method with a view to comparing the costs of both methods in general and also with respect to insurance reimbursements. The secondary objective of this study is to examine the effect of selected calculation parameters, using the sensitivity analysis to model various scenarios. The input parameter of the sensitivity analysis is represented by the time required for one patient fraction on the linear accelerator and the time the Radiology Assistant needs to prepare one complete radiation plan as part of radiotherapy planning. Furthermore, the case study calls attention to the significant impact of grant schemes directly related to the amount of write-offs included in the ABC calculation. It is the amount of these write-offs that can significantly bias the result of the overall calculation.

## 2. Materials and Methods

### 2.1. Input Data

All input data have been gleaned from the records of a regional hospital with 46 departments and total capacity of 973 beds. Employing 2708 staff members, the hospital provides medical care to approximately 460,000 patients. The range of inclusion criteria for this case study has been limited to include only information on patients with a confirmed C61 diagnosis. The reference period was the period of 2018–2019, with the cost data on the 3D-CRT technology operation referenced to 2018 and IMRT data to 2019 as the latter technology had only recently been included in the treatment routine. Both irradiation technologies used a linear accelerator (LA) supplied by Elekta Services, s. r. o. To recalculate the data and adjust them to the same basis, the input data for 2018 were discounted in a manner similar to other authors [21,31,32]. For this study, a 4% real financial discount rate was applied, as recommended by the European Commission for public investment projects co-funded from European funds. The input data were provided in CZK and, as of 31 December 2019, converted to EUR at the Czech National Bank conversion rate in effect.

The Department of Radiation Oncology made available all required information regarding its patients treated using external radiotherapy with photon beam radiation during the reference period. The basic input data include information concerning, in particular, the total hospital costs, separate radiotherapy department costs and staffing levels, pay levels, number of patients, and the volume of procedures/interventions with respect to both radiotherapy technologies. Additional economic data were gleaned from the internal Oracle Business Intelligence system and the Medicalc information system, while non-financial indicators were obtained from the Mosaiq oncology verification system. See Table 2 for a summary of the input data.

### 2.2. Description of ABC and Its Application in Radiotherapy

**Figure 1:**
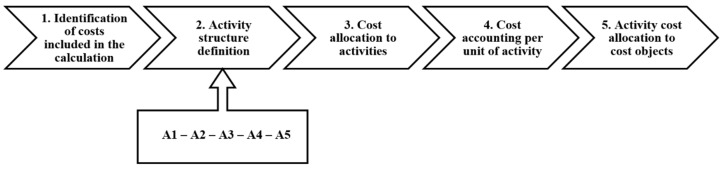
Component phases of the Activity Based Costing (ABC) method.

The default chart describing the component phases of the ABC method is based on Popesko et al. [33] recommendations and has been further supplemented by selected specific sub-phases according to Lievens et al. [29]. For a chronological description of individual ABC phases see Figure 1. A more detailed description is provided in Table 3. The Activity codes (A1–A5) are performed in Table 5.

The determination of activity unit costs (JNA) constitutes a kind of intermediate stage in the conversion of activities to cost objects, expressed as a ratio of total costs of activities to the measure of their performance/output. The denominator in the formula for JNA calculation varies according to the character of each activity (JNA1 is calculated as a ratio of total costs A1 to the number of patients). The unit costs of an activity are calculated according the following formula:

(1) $$\mathrm{JNAi}=\;\frac{\mathrm{CNAi}\;}{\mathrm{MVAi}}$$

where CNAi—Total activity costs, MVAi—Activity performance rate, JNAi—Unit of activity cost, i—activity order/number.

The labor costs of a given activity (CPA) are determined based on the following relationship:

(2) CPAi=MNAi · NMAi · Ti · P

where MNAi—number of employees participating in an activity, NMAi—gross employment costs, Ti—activity duration, i—activity order/number, P—number of patients with C61 diagnosis.

**Figure 2:**
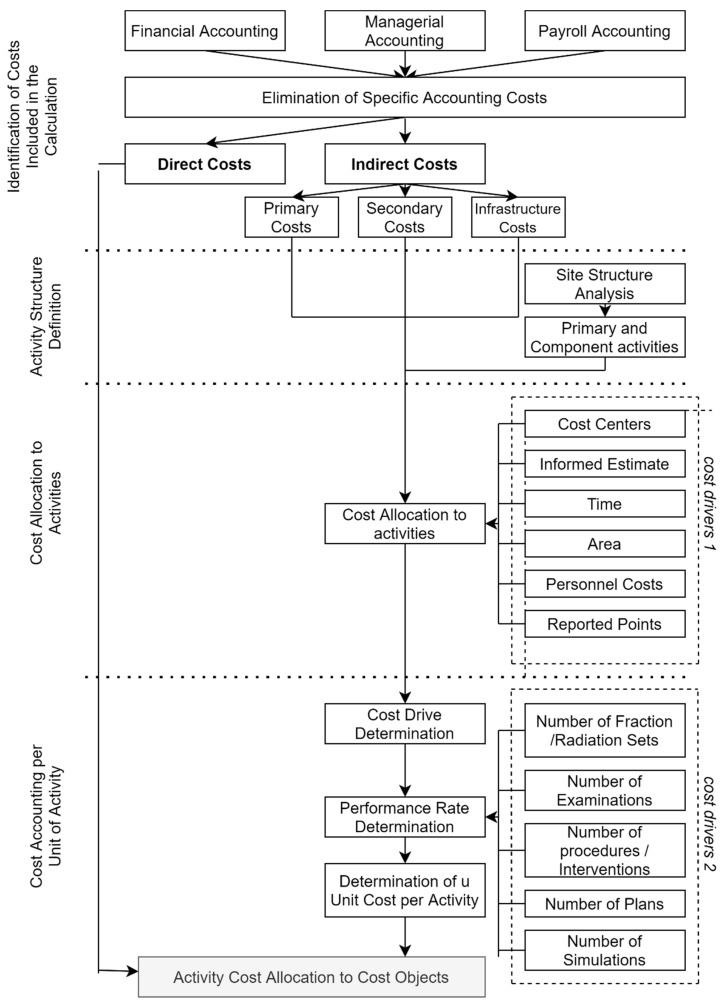
ABC process.

See the following chart in Figure 2 for a more detailed description and interconnection of actions involved in the calculation of total costs with respect to the C61 diagnosis.

## 3. Results

### 3.1. Identification of Costs Included in the Calculation

The basic input information for the ABC calculation is the cost item overview for the entire department for both reference periods, see Table 4.

### 3.2. Activity Structure Definition

Component activities were defined as part of the second phase. See Table 5 for their identification and detailed description.

Direct costs also include direct labor, further allocated based on the actual time spent on each activity. In addition, specific costs that cannot be attributed to any activity should be set aside from the identified total costs. The cost items removed for this purpose included travel expenses and the road tax as these costs would unnecessarily skew the ABC model output. Indirect costs have been assigned to individual activities based on the actual consumption ratio related to specific activities, see below. The costs are further categorized into: primary costs (Table 6), secondary costs (Table 7), and infrastructure costs (Table 6). The percentage distribution of cost items among component activities, based on the actual usage, was determined by an on-site group of experts (Head of the Comprehensive Oncology Center, Head of the Economic Department, 2 Radiology Physicists and Senior Radiology Assistant).

Furthermore, it is essential that any secondary costs be factored into the costing model (their amount having been determined based on issued invoices and the number of examinations performed at the request of the Radiation Oncology Department at specialized facilities).

### 3.3. Cost Allocation to Activities

The output of this phase is the Activity Cost Matrix developed based on suitable cost drivers. A key was used in the process of cost allocation to activities, see Table 5 and Table 6. Supporting information included the average wages of employees involved in the treatment process and the time analysis for both techniques. The duration of individual activities involved in prostate cancer treatment was measured directly at a radiation oncology facility. The working time plan for the Radiotherapy Department is 38.75 h for two-shift operation using the linear accelerators, while the weekly working time set for the rest of the department in single-shift mode is 40 h. The time analysis in relation to work performance clearly defines the number of employees and the time spent on specific activities required to perform a specific activity, see Table 8.

The labor cost matrix was calculated using the formula (2). For a summary see Table 9.

All allocated cost items were schematically entered into the cost-activity matrix with reference to cost drivers. The following table shows the cost-activity matrix for 3D-CRT and IMRT technologies (Table 10). The table includes direct, indirect as well as primary, secondary, and infrastructure costs.

**Figure 3:**
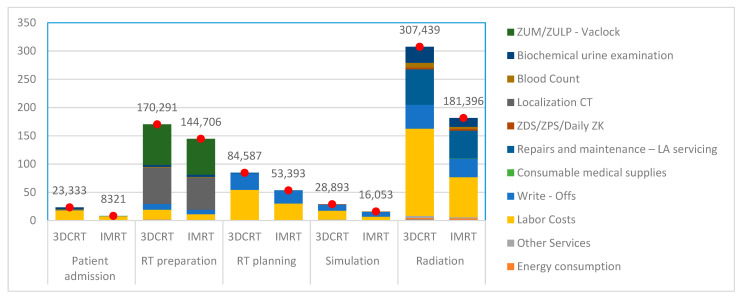
Comparison of costs of component treatment process activities for both modalities. Red dots represent the total.

The above information also lends itself to graphic representation. As clearly shown in Figure 3, the total costs of IMRT are lower with respect to all of the observed activities.

### 3.4. Activity Structure Definition

In this phase, the main output is the costing per unit of activity, see Table 11. However, the suitable cost drivers of activities (as provided in Figure 2), providing a measure of performance for each activity, were redefined prior to the calculation itself. It is also important to determine the performance rate of all activities, i.e., the exact number of cost drivers that a particular activity has created. The costs of each cost item were determined using the Formula (1).

### 3.5. Activity Cost Allocation to Cost Objects

The total costs of an activity can be determined by multiplying the unit cost of the relevant activity by the activity’s performance rate. The sum of costs of A1 to A5 component activities represents the amount of the actual costs incurred in connection with the complete radiotherapy treatment of one patient using either the 3D–CRT technology (2062 EUR) or the IMRT (intensity modulated radiotherapy) technology (1479 EUR), see Table 12.

### 3.6. Cost Balance and Insurance Reimbursements

See Table 13 for the calculated costs of both radiotherapy modalities.

IMRT costs are lower with regard to both unit and annual costs. In either case, the costs of treatment are lower than the amount of insurance reimbursements. It should be noted that the differences in reimbursements are due to the different number of patients, point value deviations and the way in which the code 43633 is reported to health insurance companies. The 43633 code per one radiation treatment was reported seven times for the 3D-CRT technology, which corresponds to the number of actual radiation fields. With the arrival of the more advanced IMRT technology and in line with recommendations of the professional society for oncology, the 43633 code per irradiation treatment began to be reported 10 times, the reason being that the process involves innumerable fields with modulated radiation beam intensity in one swing of the gantry.

### 3.7. Sensitivity Analysis

If all radiation fractions were extended or reduced by just one minute, the labor costs related to A5—Radiation would be by 16 EUR higher/lower, which in turn represents an increase/decrease in costs by 5163 EUR for the target group of 312 patients treated with 3D-CRT. With the application of IMRT, this extension or reduction in the time of radiation would amount to 12 EUR per radiation set, which would increase or decrease the overall costs of treatment of a group of 273 prostate cancer patients by 3284 EUR. See Table 14 for calculation details.

The time with respect to RT planning (A3) was analogically modulated. If the duration of the radiotherapy plan processing was 10 min longer, the costs related to 3D-CRT treatment would only increase by 441 EUR for a total of 312 patients, while the change in duration with respect to IMRT would result in an even more moderate increase of 195 EUR for a total of 273 patients. Needless to say, these values necessarily vary depending on the number of medical staff and the number of patients treated during the reference period. However, the impact of the time factor at the level of this activity is negligible in terms of costs.

The resulting cost balance is affected by the EU aid scheme (European Regional Development Fund) related to the Integrated Operational Program (IOP). The grant provided for the acquisition of two linear accelerators and a water phantom system amounted to more than 2,017,000 EUR. The grant accounts for 85% of the total purchase price, with the remaining 15% funded from the organization’s own resources. This fact must not be disregarded when using ABC costing, which shows modeling for various levels of the amount depreciated for 3D-CRT technology in 2018 (see Table 15).

The modeling can also be conducted for IMRT technology for 2019 by analogy, see Table 16.

As the results show, in the case of 100% funding from own resources, insurance reimbursements exceed the actual costs of C61 diagnosis.

## 4. Discussion

Despite the increasing incidence of both malignant and benign prostate tumors with consistently lower mortality rates over the long term, mainly due to prevention and early diagnosis, oncology treatment represents a significant portion of public funds spending due to the massive volume of new cases. The rising incidence of prostate cancer can be attributed primarily to the introduction of screening tests, early diagnosis and also to population aging, as old age is one of the main risk factors responsible for the incidence of oncological diseases. Improvements in the quality of both diagnostic and treatment methods essentially go hand in hand with the rising costs of healthcare. In the field of radiation oncology, external photon beam radiotherapy is perceived as a particularly costly approach to the treatment of oncological diseases. Thanks to the dramatic technological advances seen in radiation oncology, there has been a substantial shift in the accuracy and patient safety of target volume irradiation in recent decades, while keeping the level of treatment-related toxicity within acceptable boundaries with minimum damage to critical organs. That is why radiotherapy using linear accelerator (LA) is currently commensurate with surgical treatment and prostate cancer patients have been increasingly prone to opt for external radiotherapy due to both its non-invasive approach and highly curative effect. As the high costs of radiotherapy treatment [30] require continuous economic evaluation, coupled with adequate processing quality, there is a fundamental need for thorough cost data collection and systems for accurate costing of specific treatments [4]. This study in particular touches on the continuously debated issue of the increasing costs of radiotherapy. Moreover, these costs are not easy to interpret, mainly due to the interconnectedness of processes and the high proportion of indirect costs that need to be allocated in a sophisticated way.

This study uses the example of a regional hospital to describe the costing process for the C61 diagnosis and endeavors to identify any potential pitfalls and factors with a discernible impact on the final results of the calculation. At the same time, it seeks to highlight the discrepancy between the actual costs of diagnosis and insurance reimbursements. A similar discrepancy can be observed, for example, in the Bauer-Nilsen study [34] entitled “External Beam Radiation Therapy and Brachytherapy for Locally Advanced Cervical Cancer” or in a paper published by Ning et al. [35] with a focus on “quantifying institutional resource utilization of adjuvant brachytherapy and intensity-modulated radiation therapy for endometrial cancer via time-driven activity-based costing”. A systematic review of reimbursements and their subsequent rationalization is essential in terms of public resources.

ABC is the preferred method to estimate the costs of radiotherapy, especially when comparing radiation techniques. The application of this method was successfully published in study of Yong et al. [18], Poon et al. [36] and Ploquin and Dunscombe [37].

This study compared the two different treatment techniques for the hospital internal purposes to accommodate a request submitted by its management for the evaluation of the cost data for both of these modalities. As has already been mentioned, the clinical efficiency with respect to IMRT is beyond question, as is further corroborated through studies by Yong et al. [16], Carter et al. [19], and Hummel et al. [31]. The authors agree that the development of radiotherapeutic techniques has allowed for higher conformity of irregular target volume radiation with the possibility of dose escalation, which has demonstrable positive effects on treatment results. Zemplényi et al. [21] modeled the two radiotherapeutic modalities based on a Markov model over a 10-year period only to conclude that the IMRT technique compared to 3D-CRT is exceedingly more beneficial to the quality of life at a lower cost. Perrier et al. [38] add that the use of IGRT is also an important factor when comparing radiotherapy modalities. According to catalog prices, the additional costs of a new LU, including CBCT (Cone Beam Computed Tomography), is approximately 472,916 USD. It then depends on whether CBCT is used on a daily or weekly basis to check the patient’s position. Daily verification to limit the negative impact of radiation essentially extends the radiation time and increases the costs by as much as 43%. Due to the time extension of the entire radiation session, daily CBCT monitoring of the patient’s position increases the workload and accounts for 38% of the total labor costs, driving up the final costs of treatment by 2495 USD, whereas radiotherapy with weekly patient monitoring is estimated at 1762 USD.

In view of the selected indicators, the applied fractionation modes, including dose distribution and the length of time required to perform specific activities seem to be the most significant in terms of affecting the resulting costs of individual radiotherapy techniques. It is equally important to take into account the number of irradiated patients, which varies with the radiation technology used. The greatest time consumption and a sizable percentage can be clearly seen in relation to A5 and A3 activities. Both of these activities are, to a large extent, conducted by Radiology Assistants whose time spent on a particular procedure is predetermined. However, it is during these activities that accidental increases in time consumption are most likely to occur (e.g., due to software and hardware issues with linear accelerators, incorrect irradiator or table parameters, auxiliary IGRT equipment errors, patient or radiation plan mistakes, incorrect localization by staff members, patient re-marking, etc.). Delays on the part of the patient are often caused by inadequate bladder or rectum preparation, which is essential to ensure effective treatment and prevent unwanted movement during radiation. In the event of excessive weight loss or deterioration in the clinical condition of the patient, necessitating an interruption of the radiation set, the radiation plan, including the localization CT, should be resimulated or a new plan prepared. While the proportion of individual on-site factors is unknown, their occurrence is routine. In order to determine the way in which time consumption of the two most demanding activities affects the costs of the radiotherapy as a whole, a sensitivity analysis was carried out, modifying the time required for one patient fraction on the linear accelerator and the time the Radiology Assistant needs to prepare the complete radiation plan as part of radiotherapy planning. The results indicated that radiation time adjustment at the level of A5 will cause a relatively substantial impact on the costs (with respect to 3D-CRT and IMRT, 1 min represents an increase/decrease in costs of 5163 EUR and 3284 EUR, respectively).

The level of acute and late gastrointestinal (GI) and genitourinary(GU) tract toxicity in prostate cancer patients treated with radiotherapy was determined using RTOG/EORTC, a scoring system developed by the Radiation Therapy Oncology Group (RTOG). A sample of patients treated with IMRT/VMAT reported fewer cases of both acute GI toxicity (diarrhea, tenesmus, urgent defecation, and enterorrhagia) and GU toxicity (dysuria, urgent micturition to incontinence, nocturia, and urinary obstruction). For a more comprehensive evaluation of patient outcomes, it would be appropriate to also take into account the late responses to radiation by the surrounding tissues and organs. However, in order for the comparison to be credible, two equivalent plans would have to be created for each irradiation technique with identical radiobiological efficiency and subsequently summarized based on dose volume histograms. What is more, late gastrointestinal and genitourinary toxicity related to cancer radiotherapy treatment occurs with a delay of several years and no clinical trials pertaining to this study are yet available.

Therefore, when applying ABC costing to hospitals, it is essential that the specifics of each organization and all factors likely to influence the costing results be identified and carefully considered. These results cannot be regarded as either unequivocal or applicable to other organizations. As already mentioned, major effects include the time factor, which operates at the level of A3 and A5 activities, and the amount of own resources used to purchase the technology, reflected in the amount of depreciation included in the calculation.

The inclusion of Activity Base Costing in standard procedures as part of HB-HTA can contribute to systematic cost and economic evaluation (hospital point of view). However, the systemic implementation of the ABC method must be done in such a way so that this HB-HTA “good practice method” could be easily adopted by other hospitals (transferability), as pointed out by the EU initiative [39].

## 5. Conclusions

The costs per patient with C61 diagnosis treated using the 3D-CRT and MRT technologies amounted to 2062 EUR and 1479 EUR respectively. The annual costs of 3D-CRT (312 patients) amount to 643,344 EUR and, in the case of IMRT (273 patients), to 403,767 EUR. As the results show, IMRT appears to be the less expensive technology of the two addressed in this case study. However, the results of the sensitivity analysis also need to be taken into account. The sensitivity analysis indicates that changes in the time parameter will significantly affect the resulting calculation. This is especially noticeable with regard to the A5 activity. If all radiation fractions were extended or reduced by just one minute, the labor costs would be 5163 EUR higher/lower for the target group of 312 patients treated with 3D-CRT. With the application of IMRT, this extension or reduction in the time of radiation would increase or decrease the overall costs of treatment of a group of 273 prostate cancer patients by 3284 EUR. Another important factor affecting the overall costs related to the C61 diagnosis is the grant amount awarded to the hospital. The case study presupposes a situation wherein the grant covers 85% of the purchase cost, affecting input write-offs. Accordingly, the resulting values of the total costs with respect to C61 are lower. If the write-off amount included the full costs of the linear accelerators, the total costs of C61 would more than double. However, in the case of this medical facility, insurance reimbursements would exceed the actual costs incurred by the hospital for this diagnosis even if 100% of the funding came from its own resources.

## Figures and Tables

**Table 1:** In asymptomatic patients with a life expectancy of < 5 years + low (PSA) + low GS (Gleason score), the watchful waiting method is selected [14,15]. prostate-specific antigen

Stage	Risk	T (Extent of the Primary Tumor)	PSA	GS	Comments
1–3	Low	T1–T2a	≤10 ng/mL	≤7	in patients with a life expectancy of ≥10 years, radical prostatectomy (RAPE) or curative radiotherapy (EBRT) or brachytherapy (BRT) can be applied separately. ^1^
	Medium	T1–T2a T2b–T2c	10–20 ≤20	=7 ≤7	can be treated separately with RAPE or EBRT, EBRT can be complemented with a short-term neoadjuvant/concomitant LHRH hormone therapy of 4 to 6 months to improve the overall and tumor-specific survival rate. ^2^
	High	T3a	>20	8 and over	RAPE for selected patients only. The recommended treatment is a combination of EBRT and a long-term (2 to 3 years) or short-term (6 months) LHRH hormone therapy.
4	Very high	T3b–T4	n/a	n/a	The appropriate treatment is via hormonal manipulation (orchiectomy or LHRH analogue) in conjunction with external radiotherapy (EBRT) in selected patients (good response to androgen ablation, younger age, solitary or microscopic node metastases). ^3^

^1^ Active surveillance can be considered for a life expectancy of <10 years. ^2^ Active surveillance can be considered for a life expectancy of <10 years. ^3^ Following identification of distant M1 dissemination, hormonal manipulation (orchiectomy or LHRH analogue), second-line hormonal manipulation, chemotherapy treatment for castrate-resistant cancers, palliative surgery, palliative radiotherapy, application of bisphosphonates.

**Table 2:** Input data for radiotherapy modalities being compared—fractionation chart.

Characteristics	3D-CRT	IMRT with Rotational VMAT Mondulation
Total number of patients per department	1211	1407
Number of C61 patients	312	273
Technical equipment	RTG SIM, CT, LU, IGRT	RTG SIM, CT, LU, IGRT
Single dose applied	2 Gy per fraction	2.5 Gy per fraction
Number of radiation fractions	39	28
Fractionation mode	Standard fractionation	HART
Total radiotherapeutic dose per patient	78 Gy	70 Gy
Number of irradiated segments	7	10
Boost	sequential	simultaneous integrated (SIB)
Photon radiation energy	15 MV	6 MV
Staffing	1 KO, 3 RO, 2 RF, 6 RA, 1 JOP, 1 POP, 1 REF	1 KO, 3 RO, 2 RF, 6 RA, 1 JOP, 1 POP, 1 REF
IGRT	XVI weekly	XVI daily

**Table 3:** Description of the ABC method component phases.

Phase Nr	Phase Description
1	Total cost classification into direct and indirect costs Adjustment of cost data—bias elimination (contractual fines and sanctions, reinvoicing or adjustments)
2	Classification of costs into 3 groups: (a) primary—consumed directly by cost objects, (b) secondary—not consumed directly by a specific activity, but representing, for example, complementary diagnostic, hematological or biochemical examinations at a specialized department of the relevant healthcare organization (support activities to facilitate primary activities) (c) infrastructure activities—activities ensuring the operation of the entire department, i.e., maintenance and building administration (e.g., long-term stability tests, operational stability tests, daily instrument/device testing, electrical and gas inspections)
3	Selection of suitable cost drivers—measurable values (e.g., the number of employees participating in an activity) Work performance time analysis Measuring unit selection (e.g., m^2^) Direct assignment and determination of qualified estimates Completion of an Activity Cost Matrix (a schematic assigns calculated cost values to individual activities, thereby providing the resulting information concerning their cost structure)
4	Determination of activity cost drivers (i.e., transaction quantities, time quantities, force quantities, calculation sheets) Determination of an activity performance rate- MVAi (identifying the exact number of cost drivers created by an activity during the relevant reference period) Calculation of activity unit costs—JNA Assignment of support activity costs to primary activities—quantification of the number of secondary activity procedures/interventions required by a primary activity
5	Preparation of an overview of consumed unit costs of activities on the activity account (the number of specific activity units consumed by a cost object). Cost calculation of individual activities

**Table 4:** Radiology department cost overview (EUR).

Cost Items	Total Costs per Department	
	2018	2019
Material consumption	17,706	12,850
Energy consumption	20,120	16,541
Travel expenses	3427	2159
Other services	22,178	13,382
Labor costs	807,023	603,456
Road tax	16	13
Tangible/intangible fixed asset depreciation	422,962	321,227
Repairs and maintenance	288,701	223,063
Total costs	1,582,132	1,192,691

**Table 5:** Definition of component activities of the treatment process.

Activity Code	Activity Title	Activity Performed
A1	Patient admission	identification, evaluation of the clinical condition of the disease, patient instruction, signature of the IS with the procedure, preparation of RT documentation, making an RT appointment for the patient, data entry in Medicalc and medical documentation
A2	RT preparation	patient identification, acquiring the patient’s photograph, procedural instructions, preparation of fixatives, X-ray of the pelvis, zero point determination, location mark placement, RT report preparation, localization and CT acquisition, data export to the Monaco system, RT report printout, surface disinfection, completion of medical documentation
A3	RT planning	identification, 3D reconstruction, target volume definition, contouring, ROI plotting, dose prescription, isocenter determination, irradiation plan preparation, optimization, RT plan approval, verification, RT plan export to SIM and LU, dosimetric parameter review, RT plan printout
A4	Simulation	identification, chip ID assignment, procedural instructions, patient fixation and alignment, plotting of auxiliary structures in DDR, SIM settings, X-ray, position deviation correction, calculation of the zero position of the table, location mark placement, RT report printout, review, plan verification, export of values to LU, surface disinfection
A5	Radiation	identification, patient instruction, RT plan upload, patient fixation, zero position alignment, departure setting, XVI acquisition, online position correction, irradiation, entry in the RT report, inspection, surfaces disinfection, code reporting to health insurance companies

**Table 6:** The percentage distribution of cost items among component activities based on actual usage.

Cost Item	Patient Admission	RT Preparation	RT Planning	Simulation	Radiation
Material consumption	5%	30%	3%	20%	42%
Consumable med. Supplies ^1^	5%	20%	0%	20%	55%
Energy consumption ^2^	3%	10%	7%	10%	70%
Other services	4%	11%	5%	10%	70%
Depreciation ^3^	1%	12%	31%	12%	45%
Repairs and maintenance—LA servicing ^4^	0%	0%	2%	0%	98%
ZDS/ZPS/Daily ZK ^5^	0%	11%	0%	11%	78%

^1^ Medical material consumption—included under material consumption, with a separate estimate produced for this item. ^2^ The consumption of energy was divided according to square meters, taking into account the location of medical devices and their energy consumption. ^3^ A 15-year depreciation period based on the accounting depreciation plan is applied. ^4^ The item comes under the Repairs and Maintenance category, representing infrastructure costs. ^5^ Long-term stability test/operational stability test/daily tests.

**Table 7:** The percentage distribution of secondary costs among component activities based on actual usage.

Cost Item	Patient Admission	RT Preparation	RT Planning	Simulation	Radiation
Localization CT	0%	100%	0%	0%	0%
Blood count	10%	10%	0%	0%	80%
Biochemical urine examination	10%	10%	0%	0%	80%

**Table 8:** Intensity Modulated Radiation (IMR) and Three-Dimensional Conformal Radiation Therapy (3D-CRT) time analysis.

Activity	Position	Number of Employees		Procedure Duration	
		3D-CRT	IMRT	3D-CRT	IMRT
A1	Physician—Clinical Oncologist	1	1	35	35
	Ward Nurse	1	1	20	20
	General Nurse	1	1	15	15
	Receptionist	1	1	7	10
A2	Physician—Radiation Oncologist	1	1	35	35
	Radiology Assistant	1	1	15	15
	CT Radiology Assistant	1	2	10	5
A3	Physician—Radiation Oncologist	1	1	90	80
	Radiology Physicist	1	1	120	80
	Radiology Assistant	1	1	390	210
	JOP—verification	1	-	10	-
	Review by another RF	1	1	20	45
A4	Physician—Radiation Oncologist	1	1	25	25
	Radiology Assistant	1	1	25	25
	JOP (Technician)—inspection	1		15	15
A5	Physician—Radiation Oncologist	1	1	16	20
	Radiology Assistant	3	3	19	15
	Orderly (POP)	1	1	5	5

**Table 9:** Labor costs matrix (EUR).

Position	A1		A2		A3		A4		A5	
	3DCRT	IMRT	3DCRT	IMRT	3DCRT	IMRT	3DCRT	IMRT	3DCRT	IMRT
Physician—Clinical Oncologist	14	14	0	0	0	0	0	0	0	0
Physician—Radiation Oncologist	0	0	14	23	37	33	10	10	117	71
Ward Nurse	3	3	0	0	0	0	0	0	0	0
General Nurse	2	2	0	0	0	0	0	0	0	0
Senior RA	4	4	4	4	4	4	4	4	4	4
RA ^1^	0	0	6	8	55	30	4	4	314	180
Radiology Physicist	0	0	0	0	43	39	0	0	0	0
Technician (JOP)	0	0	0	0	2	0	2	2	0	0
Receptionist	1	1	0	0	0	0	0	0	0	0
Orderly (POP)	0	0	1	1	0	0	0	0	28	28
Cleaner	3	3	1	1	2	2	1	1	2	2
Total per patient	27	28	25	36	142	108	21	21	465	285
Total per C61 diagnosis	8370	7644	7750	9828	44,304	29,484	6552	5733	145,080	77,805

^1^ RA—Radiology Assistant.

**Table 10:** 3D-CRT and Intensity Modulated Radiation Therapy (IMRT) technology costs matrix (EUR).

Cost Items	Type of Radiation Technology	A1	A2	A3	A4	A5
Material consumption	3D-CTR	196	1178	118	785	1649
	IMRT	145	869	87	579	1217
Energy consumption	3D-CTR	134	446	312	446	3123
	IMRT	112	373	261	373	2610
Other services	3D-CTR	197	541	246	492	3443
	IMRT	121	332	151	302	2112
Labor costs	3D-CTR	17,584	17,073	53,640	15,719	154,334
	IMRT	7411	9609	29,505	5667	70,702
Write-offs	3D-CTR	628	10,983	28,991	10,983	42,206
	IMRT	485	8480	22,384	8480	32,587
Consumable medical supplies	3D-CTR	5	20	0	20	56
	IMRT	48	190	0	190	524
Repairs and maintenance —LA servicing	3D-CTR	0	0	1280	0	62,739
	IMRT	0	0	1006	0	49,280
ZDS/ZPS/Daily ZK	3D-CTR	0	448	0	448	3176
	IMRT	0	461	0	461	3269
Localization CT	3D-CTR	0	63,249	0	0	0
	IMRT	0	56,038	0	0	0
Blood count	3D-CTR	1044	1044	0	0	8349
	IMRT	0	841	0	0	3362
Biochemical urine examination	3D-CTR	3546	3546	0	0	28,365
	IMRT	0	3933	0	0	15,734
ZUM/ZULP ^1^—Vaclock	3D-CTR	0	71,763	0	0	0
	IMRT	0	63,581	0	0	0

^1^ ZUM—separately charged material; ZULP—separately charged medical preparations.

**Table 11:** Cost accounting per unit of activity.

Activity	Cost per Activity (EUR)		Cost Driver	Performance Rate		Unit Costs (EUR)	
	3D-CRT	IMRT		3D-CRT	IMRT	3D-CRT	IMRT
A1	23,333	8321	Number of patients	312	273	75	30
A2	170,291	144,706	Number of examinations	312	273	546	530
A3	84,587	53,393	Number of plans	624	410	136	130
A4	28,893	16,053	Number of simulations	312	273	93	59
A5	307,439	181,396	Number of fractions	12,168	7644	25	24

**Table 12:** Costing sheet—1 patient with C61.

Activity	Activity Unit Costs (EUR)		Cost Driver	Performance Rate		Total Costs (EUR)	
	3DCRT	IMRT		3DCRT	IMRT	3DCRT	IMRT
A1	75	30	Number of patients	1	1	75	30
A2	546	530	Number of examinations	1	1	546	530
A3	136	130	Number of plans	2	2	271	195
A4	93	59	Number of simulations	2	1	185	59
A5	25	24	Radiation set	39	28	985	664
Total						2062	1479

**Table 13:** The resulting 3D-CRT and IMRT costs balance and insurance reimbursements related to the C61 diagnosis.

Costs	Activity	3D-CRT	IMRT	Difference
Costs per patient	A1	75	30	45
	A2	546	530	16
	A3	271	195	76
	A4	185	59	126
	A5	985	664	321
	Total (EUR)	2062	1479	583
Costs per annum	Number of patients	312	273	39
	Total (EUR)	643,344	403,767	239,577
	Insurance reimbursement (EUR)	2,674,064	2,217,837	456,227
	Resulting balance (EUR)	2,030,720	1,814,070	n/a

**Table 14:** Time modulation in relation to the A5 activity.

Fraction Duration (min) 3D-CRT	Radiation 1 Patient (EUR)	Radiation 312 Patients (EUR)	19 Min Difference (EUR)	Fraction Duration (min) IMRT	Radiation 1 Patient (EUR)	Radiation 273 Patients (EUR)	15 Min Difference (EUR)
15	248	77,443	20,652	12	144	39,409	9852
16	265	82,606	15,489	13	156	42,693	6568
17	281	87,769	10 326	14	168	45,977	3284
18	298	92,932	5163	15	180	49,261	-
19	314	98,095	-	16	192	52,545	3284
20	331	103,257	5163	17	205	55,829	6568
21	348	108,420	10,326	18	217	59,113	9852
22	364	113,583	15,489	19	229	62,397	13,136
23	381	118,746	20,651	20	241	65,681	16,420
24	397	123,909	25,814	21	253	68,965	19,704

**Table 15:** Sensitivity analysis on changes to depreciated amounts included in ABC costing—3DCTR.

Own Resources Ratio for Asset Financing	Costs per 1 Patient (EUR)	Costs per 312 Patients (EUR)
15%	2062	643,344
30%	2398	748,176
45%	2734	853,008
60%	3070	957,840
75%	3406	1,062,672
100%	3965	1,237,080

**Table 16:** Sensitivity analysis on changes to depreciated amounts included in ABC costing—IMRT

Own Resources Ratio for Asset Financing	Costs per 1 Patient (EUR)	Costs per 273 Patients (EUR)
15%	1479	403,804
30%	1744	476,192
45%	2009	548,580
60%	2275	620,968
75%	2540	693,356
100%	2982	814,003

## Data Availability

Data sharing not applicable.
